# Self-assembled 2,4-dichlorophenol hydroxylase-inorganic hybrid nanoflowers with enhanced activity and stability†

**DOI:** 10.1039/c8ra02360c

**Published:** 2018-06-07

**Authors:** Xuexun Fang, Chengkai Zhang, Xue Qian, Dahai Yu

**Affiliations:** Key Laboratory for Molecular Enzymology and Engineering of Ministry of Education, College of Life Science, Jilin University 2699 Qianjin Street Changchun 130012 P. R. China yudahai@jlu.edu.cn +86-431-85155240 +86-431-85155249

## Abstract

2,4-Dichlorophenol hydroxylase (2,4-DCP hydroxylase) is a key enzyme in the degradation of 2,4-dichlorophenoxyacetic acid in the hydroxylation step in many bacteria. Our previous study demonstrated that a cold-adapted 2,4-DCP hydroxylase (tfdB-JLU) exhibits broad substrate specificity for chlorophenols, biphenyl derivatives and their homologues. However, the stability of this enzyme is not satisfactory in practical use. There have been no reports of immobilizing a cold-adapted enzyme to improve its activity and stability so far. This study for the first time reports a facile approach for the synthesis of hybrid nanoflowers (hNFs) formed from cold-adapted 2,4-dichlorophenol hydroxylase (tfdB-JLU) and Cu_3_(PO_4_)_2_·3H_2_O. The influence of experimental factors, such as the pH of the solution mixture and the enzyme and Cu^2+^ concentrations, on the activity of the prepared tfdB-JLU-hNFs is investigated. The morphologies of the tfdB-JLU-hNFs are further analyzed by SEM and TEM. Compared to the free enzyme, the tfdB-JLU-hNFs exhibit up to 162.46 ± 1.53% enhanced 2,4-dichlorophenol degradation activity when encapsulated at different enzyme concentrations. The tfdB-JLU-hNFs exhibit excellent durability with 58.34% residual activity after six successive cycles, and up to 90.58% residual activity after 20 days of storage. These results demonstrate that this multistage and hierarchical flower-like structure can effectively increase enzyme activity and stability with respect to those of the free enzyme. The satisfactory removal rate of 2,4-dichlorophenol catalyzed by tfdB-JLU-hNFs suggests that this immobilized enzyme exhibits great potential for application in bioremediation.

## Abstract 2

Highly stable and active hydroxylase-inorganic hybrid nanoflowers with great potential for application in bioremediation were obtained.

## Graphical abstract

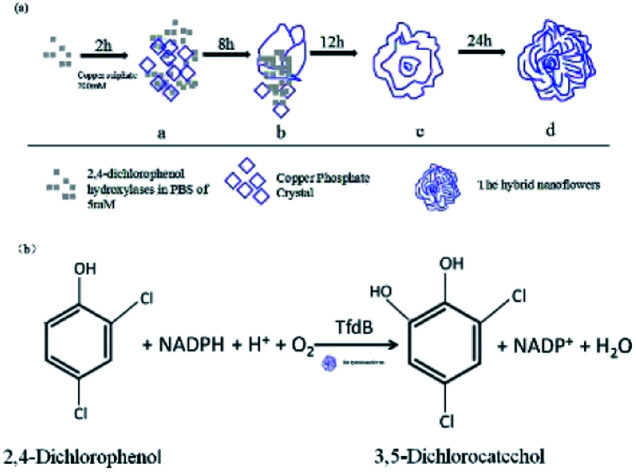

## Introduction

Chlorophenols (CPs) are extensively used to manufacture dyes, drugs, pesticides, and other industrial products.^1^ However, these substances have been a concern in the global environment because of their persistence, toxicity, and health risks.^2^ Recently, several biological methods, such as enzymatic and microbial biodegradation or transformation have been attracting great attention for removal of CPs from the environment because they are more competitive and environmentally friendly than physicochemical techniques.^3^

Enzymatic degradation is superior to the microbial biodegradation method mainly because the enzymes can tolerate concentrated CPs.^4^ Several oxidoreductase enzymes, such as 2,4-dichlorophenol hydroxylase (2,4-DCP hydroxylase; EC 1.14.13.20), peroxidase (EC 1.11.1.7), and laccase (EC 1.10.3.2), have been utilized to remove CPs through hydroxylation.^5,6^ Among these enzymes, 2,4-DCP hydroxylase (tfdB) can be effectively used to remove CPs in the hydroxylation step with the obtained products being less toxic and easily degradable during the subsequent enzymatic reaction.^7,8^ In our previous study, a new 2,4-DCP hydroxylase (tfdB-JLU) was identified in a metagenome constructed from polychlorinated biphenyl-contaminated soil. This enzyme exhibits high biodegradation ability and a wider substrate spectrum toward CPs than the previously reported tfdBs at both moderate and low temperatures.^6,9^ However, our study showed that the instability, lack of reusability and high cost of free enzyme strictly limited its practical use. tfdBs usually rapidly lose its activity in aqueous solutions and against environmental changes probably due to their susceptibility and unfavourable conformations. Thus, the recyclability and repeatability of this enzyme needs to be improved to promote its application in bioremediation.^10^ To fill this research gap, research concerning stability improvement needs to be conducted.

Immobilization of enzyme has been considered as an efficient strategy to improve enzyme stability and reusability, which not only reduces the enzyme cost but also makes them industrially feasible.^11,12^ Recently, inorganic hybrid nanoflowers (hNFs) as immobilized carrier has been widely studied in the field of immobilization for its specific hierarchical structure and functionality.^13–16^ Biomolecular including protein molecules, amino acids or DNAs have successfully been used as the synthetic template to form the hNFs with copper phosphate. Amino acid-inorganic hNFs has reported to have great potential applications in biosensors.^17^ Park *et al.* synthesized a hNFs using DNA as the organic component and the resultant hNFs not only exhibited low cytotoxicity and a high encapsulation yield but also resisted against nuclease, which would consequently be applied to safely deliver nucleic acids such as antisense oligonucleotides into cells.^18^ Recently, some new organic component such as green tea extract and *Viburnum opulus* L. extract have also been used for self-assemble of hNFs with metal ions and the obtained hNFs show potential for antimicrobial and catalytic applications.^19,20^

Among the biomoleculars used as organic component for synthesising hNFs, enzyme is of particularly interest. Ge *et al.* first reported the synthesis of organic–inorganic hNFs comprising copper phosphate (Cu_3_(PO_4_)_2_) and enzyme with greatly enhanced activities *via* a self-assembly process. The same principle has also been adopted by many other researchers.^21^ Majority studies have demonstrated that enzyme-inorganic hNFs showed highly enhanced activity, stability and selectivity in comparison with those of free enzyme.^22–27^ Also, enzyme-inorganic hNFs can be easily synthesized with very fast speed and do not require any toxic elements or very harsh conditions.^28^ Therefore, enzyme-inorganic hNFs are widely studied for the application in bioremediation, biosensing, enzymatic biofuel cells, bioanalytical devices, pharmaceutical applications and industrial biocatalysis.^29,30^

Currently, enzymes, including α-lactalbumin, carbonic anhydrase, peroxidase, glucose oxidase, laccase, lipase, and papain, have been reported to be efficiently immobilized by the hNFs system.^31–34^ However, to the best of our knowledge, most of the enzyme used are mesophilic enzyme. None of cold-adapted enzyme has been immobilized in this way.

The aim of this study is to use hNFs immobilization strategy to increase the reusability and storage stability of cold-adapted tfdB-JLU. The enzyme-inorganic hNFs material was synthesized by mixing tfdB-JLU, copper chloride aqueous solution, and phosphate buffered saline (PBS) under a certain proportion. The immobilization conditions of tfdB-JLU-hNFs were optimized. Activity and removal rate of tfdB-JLU-hNFs toward 2,4-DCP were studied. And reusability and storage stability of tfdB-JLU-hNFs were also evaluated. This study will broaden the types of enzyme that could be improved by hNFs immobilization technology.

## Experimental

### Materials

All chemicals used in the present study were of analytical grade. Copper sulfate pentahydrate, copper chloride, sodium chloride, potassium chloride and maltodextrin were purchased from Beijing Dingguo Chansheng Biotechnology Co. LTD. 2,4-Dichlorophenol (2,4-DCP) was purchased from Beijing J&K Scientific Biotechnology Co. LTD. Recombinant *Escherichia coli* DH5a containing the tfdB-JLU gene for 2,4-DCP hydroxylase expression was from our lab. BugBuster protein extraction reagent was from Novagen (Nottingham, UK).

### Measurement

The UV-Vis absorption spectra was performed on a SHIMADZU SolidSpec-3700/3700DUV. Scanning electron microscopy (SEM) was recorded on a JEOL JSM 7800F electron microscopy with primary electron energy of 5 kV and high resolution image was captured using FE-SEM, S4800 Type-II (Hitachi High Technologies Corporation). Transmission electron microscopy (TEM) was performed on a JEM2100 with electron energy of 100 kV. UV-Vis analysis was performed according to Fukumori F. And the suspension of the prepared nanoflower was assayed in absorbance at 340 nm.^35^ SEM and TEM analysis was performed according to Sun J. *et al.*^36^

### Protein expression and purification

tfdB-JLU protein was expressed and purified in accordance with our previous method.^6^

### Preparation of tfdB-JLU-hNFs

The tfdB-JLU-hNFs were synthesized as follows: mix 0.2 mL aqueous CuSO_4_ and CuCl_2_ solutions (50–250 mM) with 1 mL of PBS (0.1 M, pH 7.5) containing different concentrations of 2,4-DCP hydroxylase (0.05–1.2 mg mL^−1^ of total solution) for 24 h at room temperature. After 24 h incubation, the tfdB-JLU-hNFs (blue precipitation) was centrifuged at 12 000 rpm for 20 min at 25 °C and persisted the supernatant need to be measured by BCA protein concentration assay kit. Then the precipitation was washed three times with PBS buffer and dried by vacuum freeze-drier.

### Determination of tfdB-JLU-hNFs activity

The enzyme activity of tfdB-JLU-hNFs was assayed in accordance with our previous method.^9^ The tfdB-JLU-hNFs was incubated with 0.2 mM 2,4-DCP and 0.4 mM NADPH in 200 μl Na_2_HPO_4_/NaH_2_PO_4_ buffer at 25 °C, then the mixture solutions were measured by UV-Vis spectrophotometer (SHIMADZU3700/3700DUV, Japan) at 340 nm. One unit of enzyme activity was defined as the amount of enzyme which oxidized 1 μmol NADPH per minute at 25 °C. All the activities were performed for three times and statistical significance was determined by one-way analysis of variance (ANOVA) followed by Dunnett's test.

### Encapsulation yield of tfdB-JLU-hNFs

The encapsulation yield (EY) is defined as the ratio of the amount of immobilized enzyme to the total enzyme employed.^36^ The immobilized protein concentration is the protein in the supernatant subtracted from total protein after centrifugation, and the protein in the supernatant was measured by the BCA method using bovine serum albumin as standard. The following equation was used to calculate the EY of tfdB-JLU-hNFs:EY (%) = (*C*_T_ − *C*_S_)/*C*_T_ × 100%where EY is the encapsulation yield, *C*_T_ is the total amount of enzyme, and *C*_S_ is the supernatant enzyme content.

### Reusability and storage stability of tfdB-JLU-hNFs

Reusability of tfdB-JLU-hNFs were studied for six successive enzymatic 2,4-DCP degradation cycles at the optimum conditions. After each cycle of reaction, the tfdB-JLU-hNFs was centrifuged at 12 000 rpm, and then washed with PBS buffer (pH 7.5, 5 mM) three times and re-suspended in a fresh 2,4-DCP solution mixture to measure the immobilized enzymatic activity. Storage stabilities of the tfdB-JLU-hNFs were determined by incubating them at room temperature in PBS (pH 7.5, 5 mM) for 20 days. After every 4 days, the tfdB-JLU-hNFs were centrifuged at 12 000 rpm for 15 min, washed by PBS buffer and then assayed for residual activity. All the reusability and storage stability were performed for three times and statistical significance was determined by one-way analysis of variance (ANOVA) followed by Dunnett's test.

## Results and discussion

### Synthesis of tfdB-JLU-hNFs

**Scheme 1:**
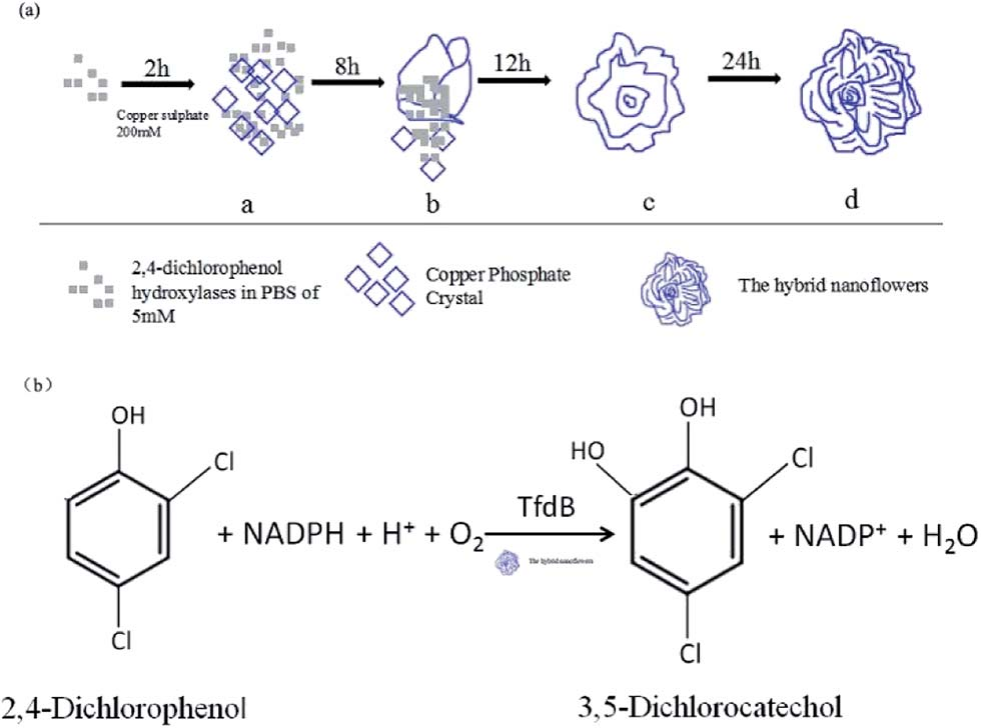
(a) The general diagrammatic illustration of synthesis of tfdB-JLU-hNFs; (b) the general illustration of the degradation mechanism for 2,4-DCP catalyzed by tfdB-JLU-hNFs.

The tfdB-JLU-hNFs were synthesized in the tubes by adding aqueous CuSO_4_ and CuCl_2_ solution with PBS (pH 7.5, 5 mM) containing different concentrations of tfdB-JLU. After 24 h of incubation at 25 °C, blue coloured precipitation of tfdB-JLU incorporated Cu_3_(PO_4_)_2_·3H_2_O and enzyme-inorganic hNFs was obtained. The general representation of tfdB-JLU-hNFs synthesis process is illustrated in Scheme 1a. The general illustration of the degradation mechanism for 2,4-DCP catalyzed by tfdB-JLU-hNFs is illustrated in Scheme 1b.

### Effect of enzyme concentration on the synthesis of tfdB-JLU-hNFs

During the synthesis of tfdB-JLU-hNFs, the enzyme formed strong complex with metal ions and proceeded through three successive steps *via* nucleation, growth and assembly into flower like structure.^37^ The nucleation step played a significant role during growth and further formation of hNFs. Copper phosphate crystals originated at Cu^2+^-binding sites for nucleation and grew to separate petals. The assembly process can be influenced by the concentration of enzyme which needs to be optimized.^38^ The effect of tfdB-JLU concentration ranging from 0.05 to 1.2 mg mL^−1^ (with respect to total solution mixture) on the synthesis of hNFs was investigated and the results were shown in Table 1. No nanoflowers were formed in the absence of enzyme. Few nanoflowers were formed at lower enzyme concentration (0.05 mg mL^−1^) due to lack of enough numbers of nucleation sites.^39,40^ The recovery activity of tfdB-JLU-hNFs were investigated with the enzyme concentration ranging from 0.05 to 1.2 mg mL^−1^ tfdB-JLU-hNFs exhibited 162.46% enhanced activity at the enzyme concentration of 1.2 mg mL^−1^. However, the amount of enzyme incorporated into hNFs dramatically decreased as the enzyme concentration increased (Table 1). At higher enzyme concentration (1.2 mg mL^−1^), EY was found to be only 6.34%. The reduction in EY could be due to saturation of inorganic component (copper phosphate) by excessive addition of enzyme.^41^ Nevertheless, at the higher EY, lower activity recovery was noticed which might be due to the hindrance of enzyme active site. These results were in accordance with the previous report.^41^ Our study also found that the presence of enzyme demonstrated profound influence on the morphology of the resultant hNFs. Loosely assembled hNFs were produced at lower enzyme concentration, while densely packed hNFs were obtained at higher concentration. Meanwhile, the average diameter of the tfdB-JLU-hNFs markedly reduced with the increase of enzyme concentration. The increase of the concentration of enzyme might lead to a corresponding increase in the number of nucleation sites, resulting in hNFs with a smaller size. The results demonstrated that enzyme worked as the size controlling agent in the synthesis process and the sizes of the hNFs were strongly dependent on enzyme concentration. Based on our results, enzyme concentration of 1.0 mg mL^−1^ was chosen as the optimal concentration for further study.

**Table 1:** The encapsulation yield and activity recovery of tfdB-JLU under different enzyme concentration (0.05–1.20 mg mL^−1^ in reaction solution) on the synthesis of tfdB-JLU-hNFs

Enzyme concentration (mg mL^−1^)	Enzyme concentration in supernatant (mg mL^−1^)	Average encapsulation yield (%)	Activity recovery (%)
0.05	0.0084 ± 0.0009	83.38 ± 1.76	69.34 ± 1.54
0.10	0.037 ± 0.0011	69.96 ± 0.61	80.45 ± 0.85
0.15	0.064 ± 0.0015	58.07 ± 1.11	84.84 ± 1.82
0.20	0.12 ± 0.0008	46.14 ± 1.52	102.28 ± 1.43
0.50	0.43 ± 0.0041	16.59 ± 0.77	125.45 ± 1.48
0.80	0.71 ± 0.0015	13.40 ± 0.39	134.59 ± 1.37
1.00	0.91 ± 0.0005	12.02 ± 0.37	152.46 ± 1.28
1.20	1.17 ± 0.0028	6.34 ± 1.07	162.46 ± 1.53

### Effect of Cu^2+^ concentration on the synthesis of tfdB-JLU-hNFs

**Fig. 1:**
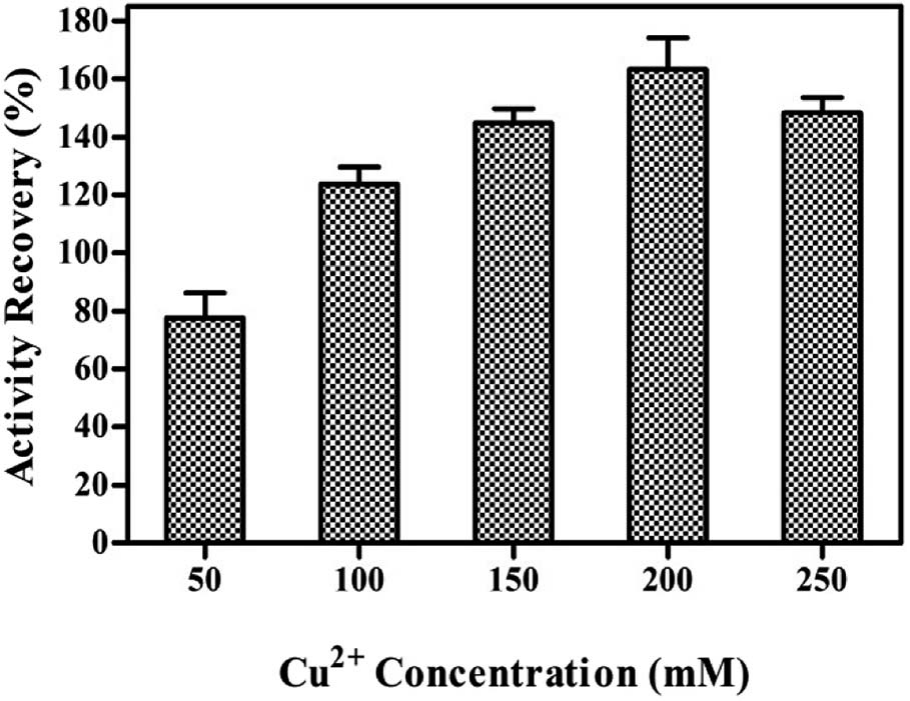
The enzyme activity recovery on synthesis the tfdB-JLU-hNFs (enzyme concentration was selected 1 mg mL^−1^) under different Cu^2+^ concentrations (50–250 mM) after incubation of 24 h in PBS buffer (5 mM, pH = 7.5) at 25 °C.

Cu^2+^ is responsible for the initiation of nanocrystals and the growth process. The effects of Cu^2+^ concentration on the synthesis of tfdB-JLU-hNFs were investigated as shown in Fig. 1. The synthesis of tfdB-JLU-hNFs was performed by dissolving tfdB-JLU (1.0 mg mL^−1^) in PBS (pH 7.5, 5 mM) at various concentrations of Cu^2+^ (50–200 mM). At early growth stage, primary crystals of Cu_3_(PO_4_)_2_·3H_2_O were formed and then embedded with tfdB-JLU predominantly *via* coordination of amide groups in the enzyme backbone.^40^ These complexes provided a location for nucleation of the primary crystal. No blue colour precipitates occurred as Cu^2+^ concentration below 50 mM, because lower copper concentration was not enough to reach the supersaturation point for the initiation of nucleation and subsequent growth of copper phosphate nanocrystals.^27^ Our result showed that 200 mM was the optimal Cu^2+^ concentration to get tfdB-JLU-hNFs with highest activity recovery.

### Growth of tfdB-JLU-hNFs

**Fig. 2:**
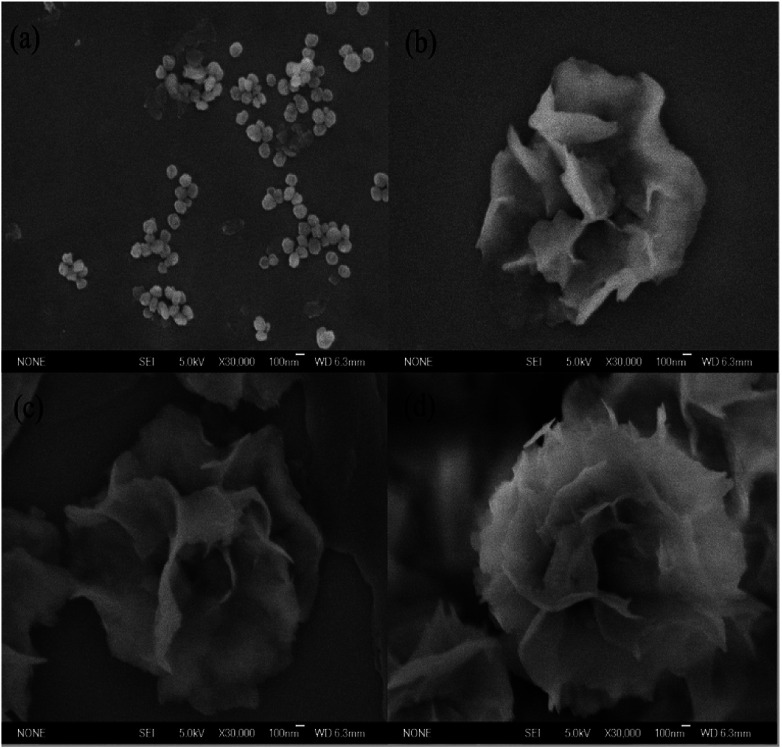
SEM images of the formation progress of tfdB-JLU-hNFs. Samples were prepared under 1 mg mL^−1^ enzyme concentration and 200 mM Cu^2+^ concentration at 25 °C in PBS buffers (5 mM, pH = 7.5) after 2 h (a), 8 h (b), 12 h (c) and 24 h (d).

The nucleation, growth and completion in nanoflowers self-assembly formation were analyzed by SEM images after incubation time of 2, 8, 12 and 24 h, respectively. The SEM images in Fig. 2 showed the formation process of the tfdB-JLU-hNFs. At an early growth step (Fig. 2a, 2 h), Cu^2+^ formed complex with few enzyme molecules primarily through coordination with amide groups of enzyme and provided a location for nucleation of primary copper phosphate crystals in 2 h.^42^ In the second step (Fig. 2b, 8 h), primary crystals phosphate of copper phosphate Cu_3_(PO_4_)_2_·3H_2_O crystals were formed with the increase of incubation time, and the separate petals were appeared. In the third growth step (Fig. 2c, 12 h), the separate petals grew into a branched flower-like structure. In the last growth step (Fig. 2d, 24 h), the branched multilayered-flower-like enzyme-inorganic hNFs structure were formed after 24 h incubation. In this growth process, enzyme induced the nucleation of the Cu_3_(PO_4_)_2_·3H_2_O crystals to form the scaffold for the petals, and also served as a “glue” to bind the petals together.

The relative activities of the obtained tfdB-JLU-hNFs were also studied after incubation time of 2, 8, 12, 24 and 72 h, respectively. It is notable that although more-hierarchical flower-like structure was obtained by further incubation from 24 to 72 h (Fig. S1†), the relative activity of the obtained tfdB-JLU-hNFs was greatly decreased from 152.46 ± 1.28 (24 h incubation) to 25.80 ± 0.17% (72 h incubation). Thus we chose 24 h instead of 72 h as optimal synthesis time. Similar result was also reported by Patel *et al.* who observed that a 24 h incubation time showed better results than 3 days incubation. Their result showed that for the synthesis of enzyme hNFs, the relative activity of the obtained immobilized enzyme was greatly decreased from 246 ± 21% (24 h incubation) to 14.4 ± 2.1% (3 days incubation) for l-arabinitol 4-dehydrogenase from *Hypocrea jecorina* and from 144 ± 16% (24 h incubation) to 17.1 ± 12% (3 days incubation) for NADH oxidase from *Streptococcus pyogenes*.^15,43^

### Characterization of tfdB-JLU-hNFs

**Fig. 3:**
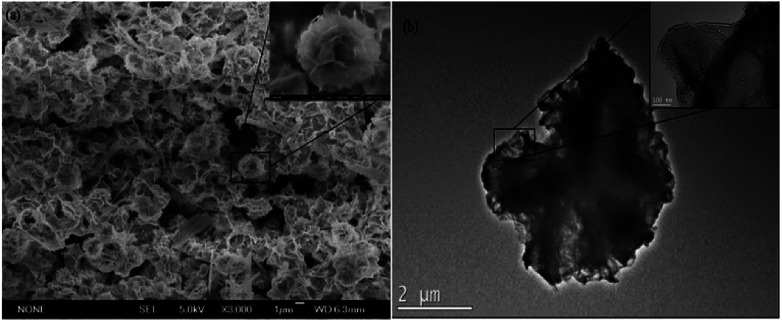
(a) SEM image of tfdB-JLU-hNFs; (b) TEM image of tfdB-JLU-hNFs. tfdB-JLU-hNFs were prepared using 1 mg mL^−1^ enzyme concentration and 200 mM Cu^2+^ concentration in PBS buffers (5 mM, pH = 7.5) at 25 °C after 24 h incubation.

The general morphologies of the tfdB-JLU-hNFs were determined by SEM and TEM. Fig. 3a presented general morphologies of tfdB-JLU-hNFs determined by SEM image (average size ∼ 1 μm), which have hierarchical structure with high-resolution images (average size ∼ 100 nm). Fig. 3b presented high-resolution TEM image (average size ∼ 2 μm) of the crystal structure of one of the petals with high-resolution images (average size ∼ 100 nm).

The result of SEM image suggested that the size of tfdB-JLU-hNFs is smaller than the normal hNFs. Our result in Fig. 2 showed that at the early stage (2 h), there were some scattered petals and primary flowers, which indicated that the process of nucleation was very short. The prolonged incubation time, however, did not result in too much change of its size although the morphologies were different. Similar results was reported by He *et al.* who observed that the diameters of the hNFs with various incubation time (0.1 h, 6 h, 24 h, and 72 h) were similar (∼7 μm).^44^ One possible reason for the small size of tfdB-JLU-hNFs might be caused by the specific structure of this enzyme.^21^ Chung *et al.* reported that the size of hNFs employed different enzyme as organic component varied considerably.^28^ This may be due to that the amide or amine groups of the employed proteins, which become nucleation sites for primary crystals of copper phosphate and serve as starting points for anisotropic growth and formation of flower-like structure, are quite different.^21^ The structure of cold-adapted enzyme is usually more flexible than that of the mesophilic enzyme, and during the immobilization, the conformation of cold-adapted enzyme might shrank and resulted in small size hNFs. Synthesis conditions have also been reported to have great impact on the size of hNFs.^15^ The small size of tfdB-JLU-hNFs might be associated with the high protein concentration we chose, which may decrease the number of nucleation sites.^21,30^

### Storage stability of tfdB-JLU-hNFs

**Fig. 4:**
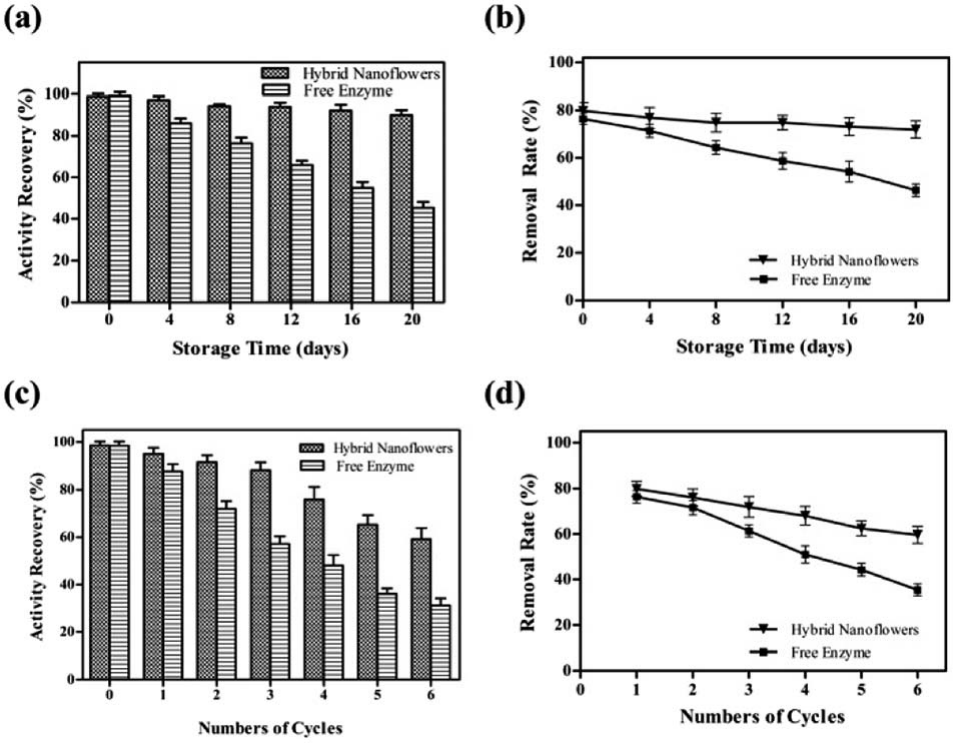
(a) Storage stability of tfdB-JLU-hNFs and free tfdB-JLU (enzyme concentration was selected 1 mg mL^−1^). Enzymes were storaged at 25 °C in PBS buffer (5 mM, pH = 7.5) after 4, 8, 12, 16, 20 days; (b) 2,4-DCP removal rate catalyzed by free tfdB-JLU and tfdB-JLU-hNFs were determined after 4, 8, 12, 16, 20 day's storage; (c) reusability of tfdB-JLU-hNFs and free tfdB-JLU (enzyme concentration was selected 1 mg mL^−1^); (d) 2,4-DCP removal rate of free tfdB-JLU and tfdB-JLU-hNFs were determined upto 6th cycle. The 100% reusability activity of free tfdB-JLU and tfdB-JLU-hNFs was determined in first cycle.

To study its storage stabilities, tfdB-JLU-hNFs was incubated in PBS buffer at room temperature. The residual enzyme activities and removal rate were measured with an interval of four days till 20 days. We found that the residual activity of free enzyme gradually reduced to only 44.70% after 20 days incubation, whereas 90.58% of initial activity of tfdB-JLU-hNFs remained under the same conditions in Fig. 4a. The increase in storage stability of the tfdB-JLU-hNFs may be attributed to minimal possible alterations in structure and enzyme active sites, which indicated the highly stable nature of hNFs in the chemical environment.^45^Fig. 4b presented high removal rate of tfdB-JLU-hNFs compared to free enzyme after 20 days incubation. tfdB-JLU-hNFs still retained 77.48% of their removal rate under the same condition.

### Reusability of tfdB-JLU-hNFs

The industrial potential of tfdB-JLU-hNFs was further determined by investigating its reusability. After each cycle, the enzyme activity of the hNFs were assayed by UV-Vis spectrophotometer. As shown in Fig. 4c, the residual enzyme activity of tfdB-JLU-hNFs was reduced to 58.34% compared to 30.26% of free enzyme after six successive cycles. Fig. 4d showed that the removal rate of 2,4-DCP by tfdB-JLU-hNFs was higher than that by free enzyme after six successive cycles. tfdB-JLU-hNFs still retained 65.26% 2,4-DCP removal ability. The mechanical damage after centrifuged and the loss of nanoflowers during repeated washing process by buffer might cause the activity decrease.^46^ However, the tfdB-JLU-hNFs still exhibited stability and excellent durability with respect to the free enzyme after multiple cycle use.

## Conclusions

In summary, this study broadens the types (cold-adapted) of enzyme that could be improved by hNFs immobilization technology. We synthesized a kind of hierarchically flower-like structured hybrid enzyme-inorganic hNFs with enhanced catalytic activity and stability. The synthesis method was simple, facile, and efficient. Additionally, reusability and storage stability of immobilized cold-adapted 2,4-DCP hydroxylase were investigated to confirm its industrial feasibility. In addition, tfdB-JLU-hNFs exhibited higher residual activity and removal rate than those of free enzyme after six successive cycles. And it exhibited higher storage stability and retained higher removal rate after 20 days incubation. This facile single immobilization strategy will have a great prospect for application in biotechnology, biomedical and environmental chemistry, and plays an important role in accelerating the degradation of CPs in the environment.

## Conflicts of interest

There are no conflicts to declare.

## Supplementary Material

RA-008-C8RA02360C-s001
