# Supplementary material for: Self-assembled 2,4-dichlorophenol hydroxylase-inorganic hybrid nanoflowers with enhanced activity and stability
Source: RSC Adv. 2018 Jun 7;8(37):20976–81. doi: 10.1039/c8ra02360c (PMC9080888; doi:10.1039/c8ra02360c)
Supplement: RA-008-C8RA02360C-s001 [file RA-008-C8RA02360C-s001.pdf]

Electronic Supplementary Material (ESI) for RSC Advances.
This journal is © The Royal Society of Chemistry 2018

Supporting information

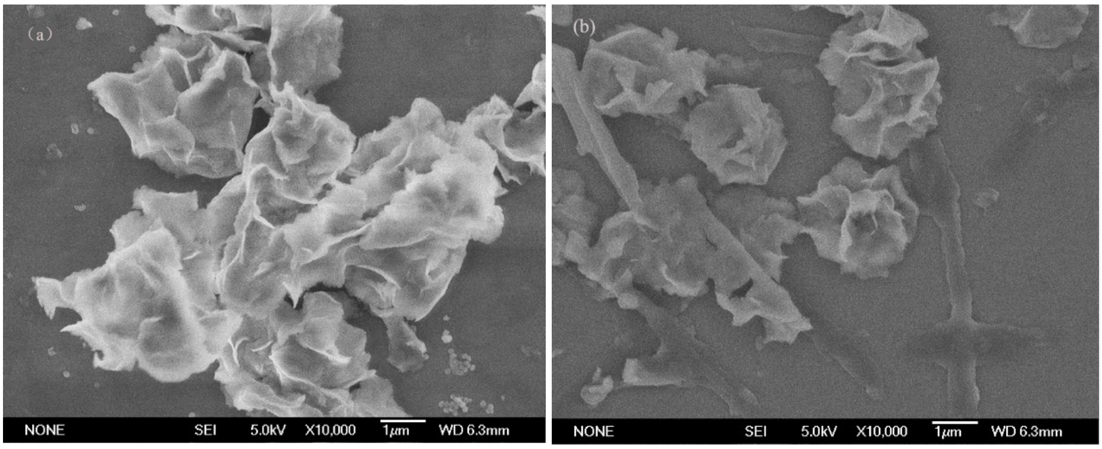

Fig. S1 SEM images of the formation progress of tfdB-JLU-hNFs. Samples were prepared under 1 mg/mL enzyme concentration and 200 mM Cu^{2+} concentration at 25°C in PBS buffers (5 mM, pH=7.5) after 24h (Fig. S1a) and 72h (Fig. S1b).

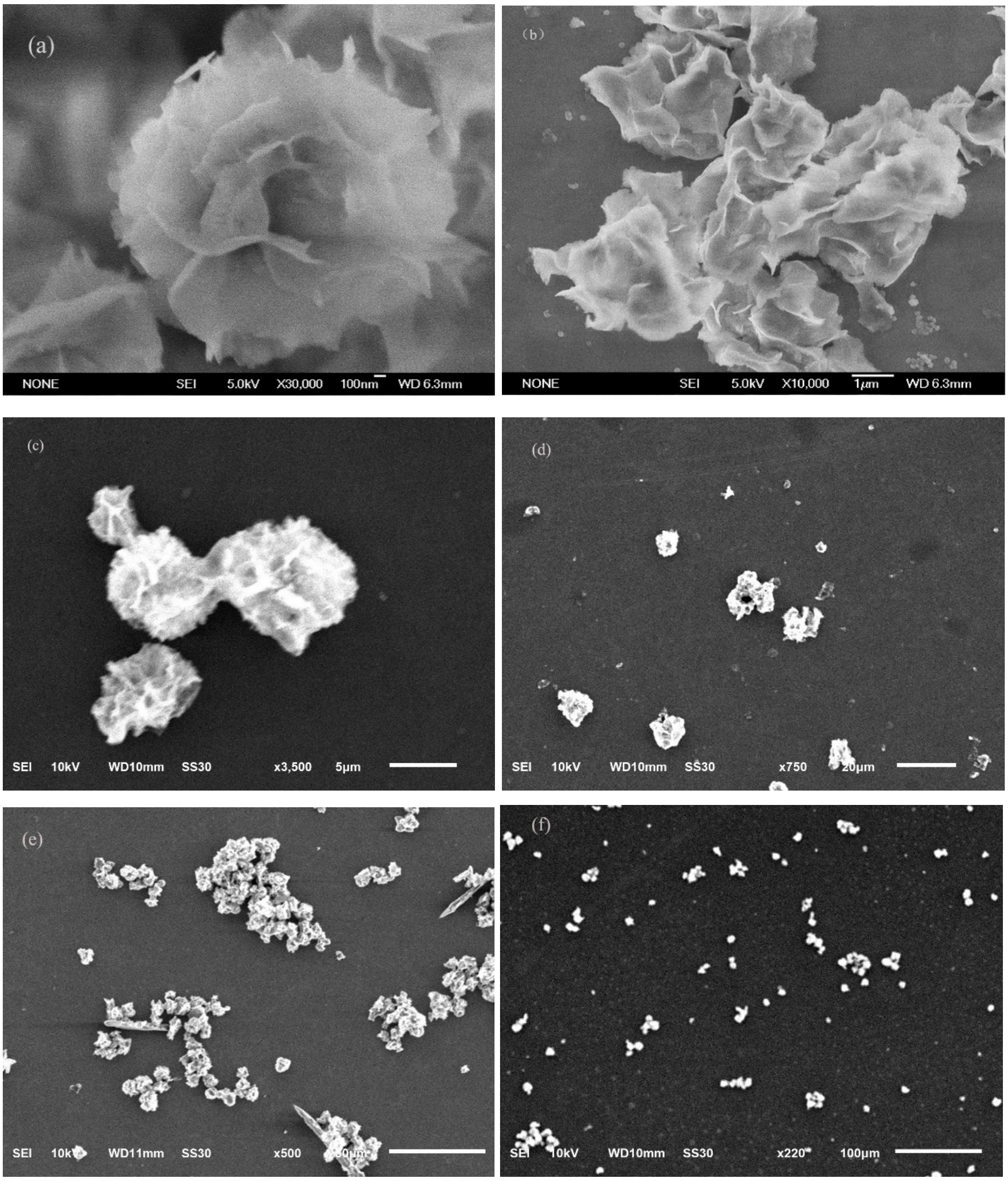

Fig. S2 SEM images of tfdB-JLU-hNFs were prepared under 1 mg/mL enzyme concentration and 200 mM Cu^{2+} concentration at 25°C in PBS buffers (5 mM, pH=7.5) in various resolutions: (a) 100 nm; (b) 1 μm; (c) 5 μm; (d) 20 μm; (e) 50 μm; (f) 100 μm.
